# Genetic analysis of the interaction between the N- and C-terminal halves of UNC-112 (kindlin)

**DOI:** 10.17912/micropub.biology.000342

**Published:** 2020-12-17

**Authors:** Hiroshi Qadota, Yating Luo, Andres F Oberhauser, Guy M Benian

**Affiliations:** 1 Department of Pathology, Emory University, Atlanta, Georgia; 2 Department of Neuroscience, Cell Biology & Anatomy, Sealy Center for Structural Biology and Molecular Biophysics, University of Texas Medical Branch, Galveston, Texas

**Figure 1. Missense mutations in the C-terminal half of UNC-112 restore binding to the N-terminal half of UNC-112 containing mutations that normally would not allow binding using the yeast two hybrid system:**
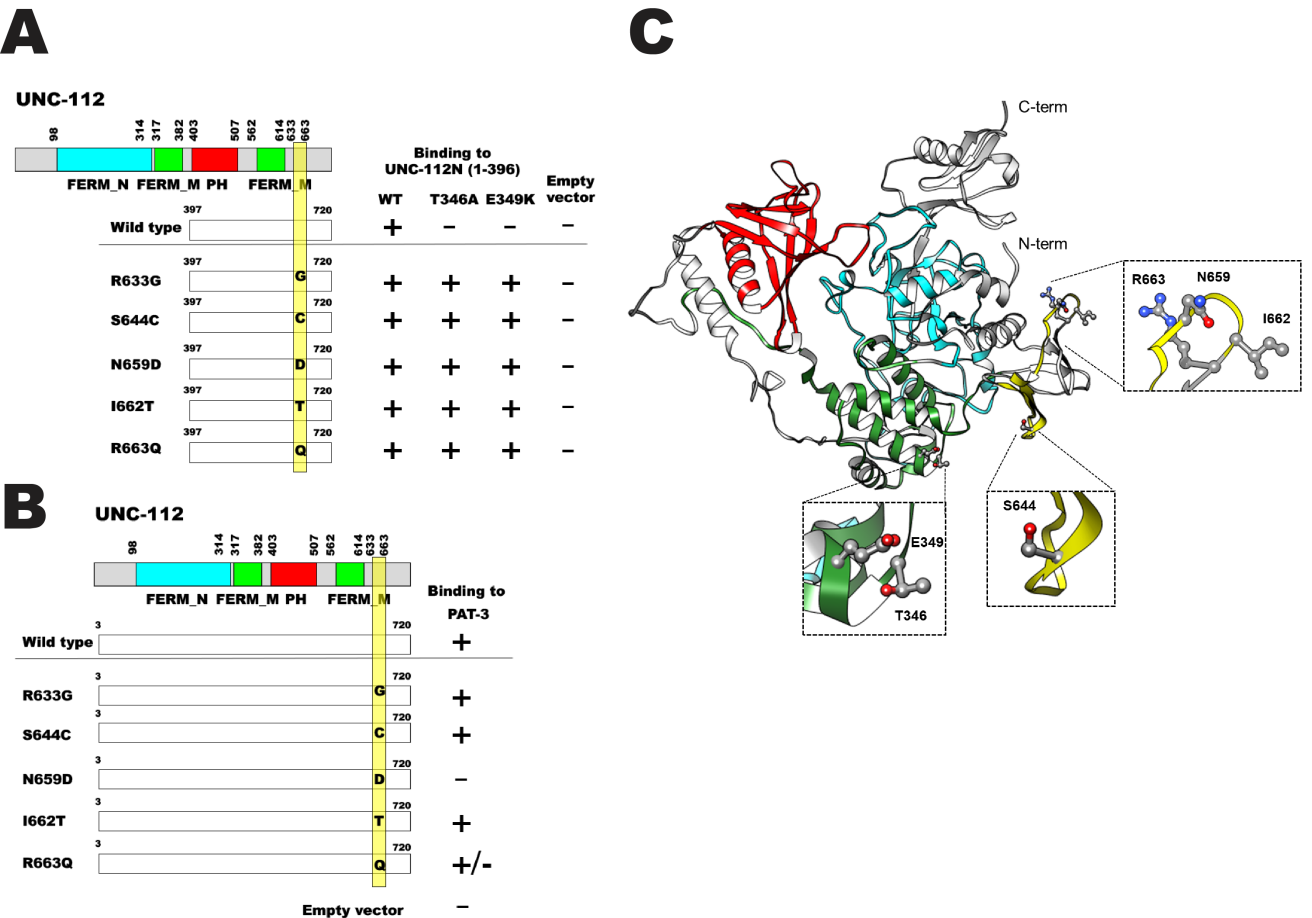
**A and B.** Schematic representation of domains in UNC-112 (kindlin), location of mutations, and results of yeast two hybrid assays.FERM_N, FERM_M, and PH are domains predicted by PFAM. Numbers indicate amino acid residue numbers in UNC-112. + represents growth on His- plate and Ade- plate. – represents no growth on either His- plate or Ade- plate. +/- represents growth only on His- plate. Empty vector refers to the empty prey vector. Yellow bar represents the 30 residue region containing the suppressor mutations. **A.** Wild type UNC-112 C-terminal half cannot bind to UNC-112 N-terminal half containing T346A or E349K. UNC-112 C-terminal half with R633G, S644C, N659D, I662T, or R663Q can bind to UNC-112 N-terminal half with T346A or E349K. **B**. Nearly full-length UNC-112 (3-720) as wild type, R633G, S644C, I662T or R663Q, but not N659D can bind to the cytoplasmic tail of PAT-3 (beta-integrin). **C.** Structure of UNC-112 based on the human kindlin-3 3D structure (PDB: 7C3M) (Bu *et al.*, 2020) modelled with Swiss-Model (Waterhouse *et al.*, 2018), and showing corresponding mutated residues. The color scheme matches the colors of the domains in the schematics of parts A and B.

## Description

*C. elegans* UNC-112 (kindlin) is required for muscle sarcomere assembly (Rogalski *et al.* 2000), and is part of a conserved four protein complex that associates with the cytoplasmic tail of integrin at the base of muscle integrin adhesion complexes (Mackinnon *et al.* 2002; Lin *et al.* 2003; Norman *et al.* 2007; Qadota *et al.* 2014). UNC-112 consists of 720 amino acids and contains FERM and PH domains. UNC-112 N-terminal half (1-396 aa) can bind to UNC-112 C-terminal half (397-720 aa), and this interaction is inhibited by the association of PAT-4 (integrin linked kinase, ILK) to the UNC-112 N-terminal half (Qadota *et al.* 2012). The UNC-112 N-terminal half with either T346A or E349K mutations cannot bind to the UNC-112 C-terminal half, but can still bind to PAT-4 (ILK) (Qadota *et al.* 2012). To elucidate the molecular mechanism by which the UNC-112 N- and C-terminal halves interact genetically, we identified suppressor mutations in the UNC-112 C-terminal half that restore the ability of the UNC-112 C-terminal half to bind to the UNC-112 N-terminal half with T346A or E349K. We introduced random mutations into the UNC-112 C-terminal half (by error-prone PCR), and assayed for binding to the UNC-112 N-terminal half with T346A or E349K. From screening using the yeast two hybrid system and DNA sequencing of suppressor clones, we identified 5 single amino acid changes; R633G, S644C, N659D, I662T, and R663Q. S644C and R663Q were identified from screening with the UNC-112 N-terminal half containing T346A, and R633G, N659D, and I662T were identified from screening with the UNC-112 N-terminal half containing E349K. The wild type and all 5 suppressor clones failed to activate growth with an empty prey vector. Our results are summarized in Figure 1A. The UNC-112 C-terminal half with S644C or R663Q can bind to UNC-112 N-terminal half with T346A as expected from screening (see Methods), but also can bind to the UNC-112 N-terminal half with E349K. The UNC-112 C-terminal half with R633G, N659D, or I662T can bind to the UNC-112 N-terminal half with E349K as expected and but can also bind to UNC-112 N-terminal half with T346A. Interestingly, these 5 single amino acid changes are located within a 30 amino acid long segment (633 to 663; yellow bar in Figure 1). We speculate that these 30 amino acid residues of UNC-112 C-terminal half form a “Pocket” corresponding to the T346-E349 region of UNC-112 N-terminal half as a “Key”. We have shown previously, that only full-length UNC-112 can bind to cytoplasmic tail of PAT-3 (beta-integrin) by yeast two hybrid and biochemical methods (Qadota *et al.* 2012). Full length UNC-112 singly harboring the 5 suppressor mutations were tested for binding to the cytoplasmic tail of PAT-3. We found that 4 of 5 suppressors (R633G, S644C, I662T, and R663Q) could still bind to PAT-3, but full length UNC-112 with N659D could not bind (Figure 1B), suggesting that N659D causes a severe conformational change.

In contrast to *C. elegans*, which has one kindlin (UNC-112), humans have three kindlins, each encoded by a separate gene (Meves *et al.* 2009). Alignment of nematode and human kindlins reveals that both of the residues mutated in the N-terminal half of UNC-112 that block its interaction with the C-terminal half are conserved in all three human kindlins. Of the five suppressor mutations, S644 is not conserved, but R633 and N659 are absolutely conserved, I662 is conserved except there is a T at this position in kindlin-1, and R663 is conserved except that there is a K at this position in kindlin-2. The only crystal structure available for a kindlin is for human kindlin-3 (Sun *et al.* 2020; Bu *et al.*, 2020). Based on this structure, we created a homology model of UNC-112 and placed the mutations described in Figure 1A on this model. Inspection of the model (Figure 1B) shows that T346 and E349 are in one cluster, that N659, I662 and R663 are in a different cluster, and S644 is isolated. Because T346/E349 and S644, N659, I662 and R663, are not in close proximity to each other, this analysis does not provide insight into how the suppression occurs. However, there are several possibilities to explain this: (a) We are considering a model and not a real structure for UNC-112. (b) The crystal structure and model are static and do not take into account possible structural flexibility or the existence of other conformations. For example, it is possible that upon binding of PAT-4 (ILK) to the N-terminal half of UNC-112, a conformational change occurs that brings our set of residues close together so that they may interact. Unfortunately, this possibility cannot be explored as a co-crystal structure of a UNC-112/PAT-4 or kindlin/ILK complex has not yet been reported.

## Methods

Random mutagenesis using PCR and screening for interactions using the yeast two hybrid method were performed as described previously (Miller *et al.* 2006; Qadota *et al.* 2012). Briefly, the UNC-112 N-terminal half was cloned into pGAD-C1 (prey plasmid), and the UNC-112 C-terminal half was cloned into pGBKT7 (bait plasmid). For random mutagenesis using PCR, two primers (TAATACGACTCACTATAGGGC and TAAGAGTCACTTTAAAATTTGTAT) were used. PCR amplified fragments of UNC-112 C-terminal half, digested pGBKT7, and pGAD-UNC-112 N-terminal half were introduced into yeast two hybrid host strain (PJ69-4A), then His+ colonies were selected. After checking for growth on Ade- plates, colonies containing pGBKT7-UNC-112 C-terminal fragment plasmids were grown in liquid media, and total yeast DNA was isolated, and transformed into XL1 Blue bacteria. LB + kanamycin plates were used to select for the growth of bacteria containing pGBKT7-UNC-112 C-terminal fragments; note that the pGAD-UNC-112 N-terminal half plasmids contains an ampicillin resistance marker. Seven such “suppressor clones” were isolated from the screening of UNC-112 N-terminal half containing the T346A mutation, and 12 suppressor clones were isolated from screening of UNC-112 N-terminal half containing the E349K mutation. DNA sequencing revealed that 10, among a total 19 clones, contained premature stop codons. All of these stop codons occurred near the N-terminus of UNC-112C and when translated would result in GAL4 DNA binding domains fused to only 14-25 amino acids from UNC-112C. Moreover, 4 of 10 of these showed non-specific growth (i.e. with an empty prey vector). For these reasons, we decided not to study them further. Among the remaining 9 clones, 3 clones contained single amino acid changes; S664C and R663Q from the screening of UNC-112 N-terminal with T346A, N659D from the screening of UNC-112 N-terminal half with E349K. The other 6 clones from screening with the UNC-112 N-terminal half containing E349K, had two or more mutations, three of which had R633G and three of which had I662T. We created UNC-112 C-terminal half with single mutations of R633G and I662T by site-directed mutagenesis, and then tested for binding to the UNC-112 N-terminal half with E349K. Since both mutations showed binding to UNC-112 N-terminal half with E349K, we concluded that R633G or I662T are responsible for suppression in the clones containing multiple mutations. For UNC-112 protein structure modeling, the SWISSMODEL (Waterhouse *et al.*, 2018) and Phyre2 (Kelley LA *et al.*, 2015) online tools were used. Human kindlin-3 (7C3M.pdb; Bu *et al.* 2020) was used as reference crystal structure. Molecular graphics were generated by using Chimera (Pettersen *et al.*, 2004).
